# Meta-Analysis of the Efficacy of Rapid Rehabilitation Surgical Nursing in Lumbar Disc Herniation

**DOI:** 10.3390/healthcare12222256

**Published:** 2024-11-13

**Authors:** Hongchao Duan, Jun Wang, Dan Liang, Huan Liu, Feihong Sun, Chunyuan Li, Fengzeng Jian

**Affiliations:** Department of Neurosurgery, Xuanwu Hospital Capital Medical University, Beijing 100053, China; duanhongchao@xwhosp.org (H.D.); wangjun@xwhosp.org (J.W.); liangdan@xwhosp.org (D.L.); liuhuan@xwhosp.org (H.L.); sunfeihong@xwhosp.org (F.S.); lichunyuan_dr@xwhosp.org (C.L.)

**Keywords:** rapid rehabilitation surgical nursing, lumbar disc herniation, meta-analysis, intervertebral disc displacement

## Abstract

**Background:** Lumbar disc herniation (LDH) is a common cause of lower back pain and radiculopathy. In recent years, the enhanced recovery after surgery (ERAS) concept has been increasingly applied in orthopedics and gastrointestinal surgery. **Purpose:** To investigate the effect of using rapid rehabilitation surgical care for lumbar disc herniation by meta-analysis. **Data source:** Google Scholar, PubMed Medical, Cochrane and Embase databases were used for the analysis. **Research selection:** An initial search yielded a total of 322 relevant articles. Duplicate pieces of literature were screened using Endnote. In addition, non-randomized controlled trials and studies with a sample size of less than 30 were excluded. A total of seven papers were included in this study. **Main outcomes:** The Rapid Rehabilitation Surgical Nursing (RRSN) group showed significantly higher patient satisfaction (RR = 1.24; 95% CI: 1.06, 1.26; *p* < 0.01) and self-assessed health (Total MD = 5.67; 95% CI: 4.27, 7.06; *p* < 0.01) compared to the Normal Nursing (NN) group. Pain levels (MD = −0.66; 95% CI: −0.97, −0.36; *p* < 0.01), disability levels (MD = −18.64; 95% CI: −32.53, −4.76; *p* < 0.01), anxiety risk (SAS-MD = −4.33; 95% CI: −6.23, −2.44; *p* < 0.01), and depression risk (SDS-MD = −4.29; 95% CI: −7.50, −1.07; *p* < 0.01) were significantly lower in the RRSN group compared to the NN group. According to the GRADE classification, the certainty for patient satisfaction is high, while the certainty for post-care pain, functional capacity, risk of psychological disorders, and self-assessed health status is moderate. **Conclusions:** Rapid recovery surgical nursing can significantly improve postoperative recovery of lumbar disc herniation, increase patient satisfaction, reduce the risk of psychological disorders, improve lumbar function, and alleviate patient pain.

## 1. Introduction

Lumbar disc herniation (LDH) is a common disease in orthopedics, with a high incidence rate, which is more common in clinics, especially in the L4~S1 intervertebral disc [1]. The pathogenesis of lumbar disc herniation is that after the degenerative change of the lumbar spine, the annulus fibrosus of the intervertebral disc is ruptured under the action of external force, so that the nucleus pulposus tissue protrudes from the rupture, thereby compressing or stimulating adjacent nerve roots, leading to non-specific inflammatory reactions such as edema, congestion and tissue degeneration of nerve roots and the surrounding tissues caused by chemicals [2,3].

Most patients with lumbar disc herniation can be relieved by conservative treatment without surgical treatment, and patients with poor conservative treatment need surgical treatment [4]. In recent years, the incidence of lumbar disc herniation is increasing year by year. However, the development of technology has also seen a diversification of surgical treatment methods, the common methods being percutaneous lumbar discectomy, percutaneous laser disc decompression, percutaneous ozone gas injection into the intervertebral disc, posterior discectomy under intervertebral disc endoscope and so on [4,5,6].

Surgery is just the first step, and postoperative care is very important after surgery. The course of rapid rehabilitation nursing after surgery for lumbar disc herniation is often long, and patients suffer from the disease repeatedly, which makes it easy to produce anxiety and tension [7]. Previous studies have proved that rapid rehabilitation surgery is an important perioperative nursing method for LDH patients undergoing minimally invasive spinal surgery [4,8]. Targeted nursing can reduce surgical stress, relieve postoperative pain and promote postoperative rehabilitation. As an effective nursing mode in clinics, at present, rapid rehabilitation nursing fully demonstrates the concept of people-oriented nursing [9,10]. It is a more comprehensive, targeted and personalized nursing method based on routine nursing, and follows the principles of “rapid rehabilitation”, “active participation” and “step by step” [11]. According to patients’ different conditions, the rehabilitation plan should be made, and the patients should be given corresponding knowledge education and surgical condition education before, after and after discharge, together with corresponding psychological nursing intervention, simulated guidance and exercise, and rehabilitation guidance after discharge, which can effectively promote the recovery speed of patients [12].

Pain nursing in rapid rehabilitation surgery can reduce patients’ pain so that patients can actively cope with later nursing. Under the mode of rapid rehabilitation nursing intervention, psychological counseling should be given in time after admission, so that patients can correctly understand the progress of the disease and cooperate with the treatment [13].

## 2. Methods

### 2.1. Study Design

This study was conducted to evaluate the effect of Rapid Rehabilitation Surgical Nursing (RRSN) on patients undergoing Lumbar Disc Herniation (LDH) surgery. According to the “Preferred Reporting Items for Systemic reviews and Meta-analyses” guidelines. PubMed, Google Scholar, Embase, Web of Science, and Cochrane Central Register of Controlled Trials databases were used for the literature retrieval. The keywords used for the literature search include, “intervertebral disc displacement” or “intervertebral disc degeneration” or “lumbar disc” or “intervertebral disc disease” and “rehabilitation surgical nursing” or “nursing”, and all articles from database establishment to publication in December 2023 are considered.

### 2.2. Evaluation

The primary outcome measure was the satisfaction of LDH patients who received surgical treatment and different forms of care. Secondary outcome measures included patient pain levels, activity ability, psychological disease risk, and self-assessed health outcomes.

### 2.3. Literature Inclusion and Exclusion Criteria

Inclusion criteria of the literature:

(1) Patients with a clinical diagnosis of LDH were studied;
(2) The clinical trials conducted in all pieces of literature should follow the principle of randomized control trial (RCT), and there is no statistical difference in general data among patients in each group;
(3) The number of patients in each group in all pieces of literature is not less than 30;
(4) The patients who participated in the experiment in all pieces of literature were between 18 and 80 years old;
(5) In all pieces of literature, the nursing method used in the intervention group was rapid rehabilitation surgical nursing, whereas the comparator groups received either standard postoperative care or another form of enhanced recovery pathway not defined as RRSN.

Exclusion criteria of the literature:

(1) Any literature without clinical trials, such as review and case analysis;
(2) Any literature published repeatedly;
(3) Clinical trial articles or documents with incomplete data;
(4) Unpublished pieces of literature;
(5) Any literature with too small a sample size in clinical trials;
(6) In the clinical trial grouping, the intervention measures of the experimental group did not involve any literature on evidence-based nursing;
(7) The literature that the patients who participated in the clinical trial were too young or too old.

### 2.4. Literature Screening and Data Extraction

Initial screening: keyword combinations, search on major literature platforms initially, collect qualified documents, classify and sort out the collected documents and related data with the help of Excel, remove the same documents, preliminarily consult the remaining topics and abstracts collected, remove the related documents with great differences, read through the screened documents, and exclude the documents that do not meet the research standards according to the standards of document acceptance and arrangement, and record the number and reasons of the documents.

Secondary screening: conducted independently by two researchers, the collected documents were further screened and extracted according to the above criteria for inclusion and exclusion of documents, and the number and reasons for excluded documents were recorded at the same time.

Three lots of screening: when the above researchers’ suggestions for inclusion cannot be unified, another researcher will independently read and coordinate to solve the problem of literature screening.

### 2.5. Literature Quality Evaluation

Evaluate the bias of all the screened documents, mainly through Cochrane-related tools, and evaluate the quality of all the screened documents, including (1) selection, that is, judging whether the control experiment is random or not according to the situation of all the screened documents and whether the included documents mention the specific way of distribution and concealment. (2) Implementation is a double-blind test of whether all the screened documents are researchers and subjects. (3) Measurement is to judge whether the researchers know the outcome in all the screened documents, so as to avoid the result deviation caused by the blindness of the outcome. (4) Follow-up is to evaluate the incompleteness and completeness of all the screened literature test results, including missing and screened data. (5) Reporting is to evaluate the selective reporting tendency of all the screened documents, so as to avoid biased data due to publication advantages. (6) Others, that is, other possible biased factors, are referred to according to all the screened documents.

### 2.6. Statistical Methods

To correct for potential errors due to multiple comparisons, we applied the Bonferroni correction method to evaluate the statistical significance of all results in this study. According to the corrected standard, results are considered statistically significant only if the *p*-value is less than 0.05/5. With the help of RevMan 5.4 software tool, meta-analysis was made on all the filtered Meta. X^2^ test is used to process the control experimental data in all the screened documents, and the heterogeneity of the collected experimental data is evaluated by I^2^. If *p* > 0.01 and I^2^ < 50%, there is no difference in the data in all the screened documents, and the fixed effect model can be used to combine and analyze the control experimental data in all the screened documents. If *p* < 0.01 and I^2^ > 50%, the source of data heterogeneity needs to be analyzed. Carry out relevant grouping interventions. If the value is still too large, make correlation analysis of the data through random effect model, analyze the effect statistics through odds ratio (OR), estimate the interval through 95% confidence interval, exclude the clinical studies used in the literature for not less than 5 times, make sensitivity analysis according to the change of the results, and then analyze the stability of the data results.

## 3. Results

### 3.1. Literature Selection

**Figure 1:**
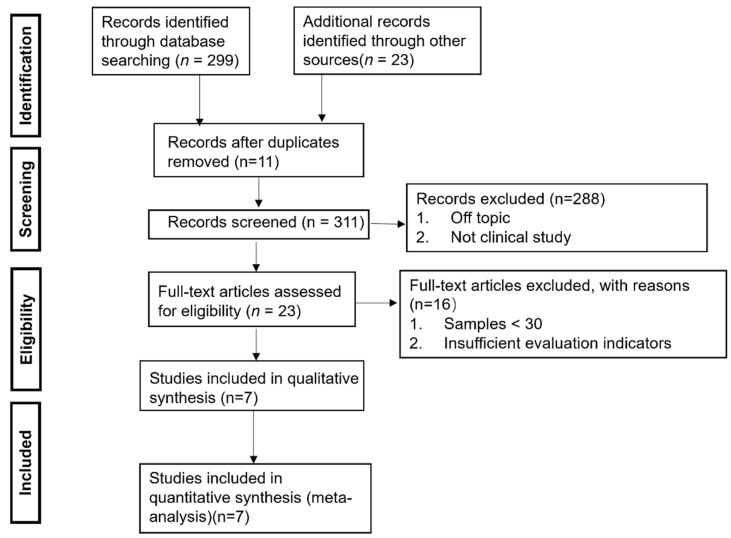
Reporting Items for Systematic Reviews and Meta-Analysis (PRISMA) flow diagram for the inclusion of studies.

A total of 322 related documents were retrieved by preliminary search. After excluding the same documents, 311 documents were left. After a preliminary reading of the remaining topics and abstracts, 288 documents were left. And after reading through the remaining documents, 16 documents were left. Papers were selected using the Preferred Reporting Items for Systematic Reviews and Meta-Analyses (PRISMA) flowchart (Figure 1).

### 3.2. Study Characteristics

A total of 795 samples were included in the seven studies, with a total of 395 cases in the control group (C, number of samples of control group) and 400 cases in the treatment group (T, number of samples of treatment group) (Table 1).

### 3.3. Risk of Bias

**Figure 2:**
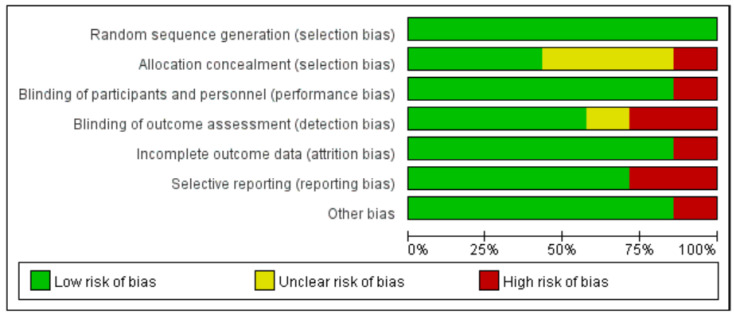
The assessment of the risk of bias in studies evaluating the effects of Rapid Rehabilitation Surgical Nursing on patients undergoing surgery for Lumbar Disc Herniation.

In this study, we used Cochrane Risk of Bias Tool 1 (RoB1) to assess the risk of bias in the included studies. While the updated Cochrane Risk of Bias Tool 2 (RoB2) is available and recommended for new studies, RoB1 has been widely used and validated in previous meta-analyses [21], providing a consistent approach for comparing results with earlier studies. However, we acknowledge the limitations of RoB1 and will consider using RoB2 in future research to ensure the most up-to-date and comprehensive assessment of the risk of bias. A risk of bias evaluation was conducted on the seven included pieces of literature, and the results showed (Figure 2) that the main source of risk in the selection bias of the included studies was allocation consideration, which may be due to the fact that most hospitals require patients to sign an informed consent form during the clinical trial implementation process. Secondly, detection bias also carries a high risk, and most studies find it difficult to achieve the binding of outcomes during detection. Based on individual risk of bias assessments, a large number of studies exhibit selection bias issues, primarily due to inadequate allocation concealment. Specifically, “random sequence generation” was found to have a 100% low risk of selection bias. “Allocation concealment” was found to have a 45% low risk of selection bias, a 40% unclear risk of selection bias, and a 15% high risk of selection bias. “Blinding of participants and personnel” was found to have an 85% low risk of performance bias and a 15% high risk of performance bias. “Blinding of outcome assessment” was found to have a 60% low risk of detection bias, a 10% unclear risk of detection bias, and a 30% high risk of detection bias. “Incomplete outcome” was found to have an 85% low risk of attrition bias and a 15% high risk of attrition bias. “Selective reporting” was found to have a 70% low risk of reporting bias and a 25% high risk of reporting bias. Among other types of bias, 85% were assessed as low risk and 15% as high risk.

### 3.4. Outcomes

To ensure the transparency and comparability of the study results, we primarily used post-intervention measurement values when calculating effect sizes. Although using the difference values before and after intervention might more accurately reflect the intervention effects, due to the lack of baseline data or non-significant baseline differences in some of the included studies, we were unable to obtain complete pre–post difference data for all studies. Therefore, we chose to use post-intervention measurement values for the analysis.

#### 3.4.1. Evaluation of Satisfaction After Nursing

A total of six studies evaluated patient satisfaction after receiving NN or RRSN. The satisfaction level also indirectly reflects the patient’s evaluation of the degree of rehabilitation. The evaluation method is in the form of a survey, mainly based on satisfaction level. Patients who are selected as “very satisfied” or “satisfied” are classified as having good postoperative recovery and analyzed.

**Figure 3:**
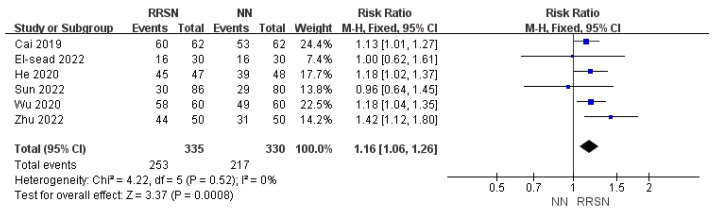
Comparison of patient satisfaction between Rapid Rehabilitation Surgical Nursing and Normal Nursing by forest plot [14,15,16,17,18,20]. Note: RRSN: Rapid Rehabilitation Surgical Nursing; NN: Normal Nursing; CI: Confidence interval. Effect size estimates are depicted by filled squares, with horizontal whiskers corresponding to 95% CIs. The filled diamond indicates the overall mean effect size.

Regarding the assessment of patient satisfaction following NN or RRSN, due to consistent results across multiple studies, we initially rated the evidence as “high”. Upon closer inspection, we found low heterogeneity (I^2^ = 0%, *p* = 0.52), and fixed-effects model analysis showed that patient satisfaction in the RRSN group was 1.16 times that of the NN group, with an RR of 1.24; 95% CI: 1.06–1.26; *p* < 0.01 (Z = 3.37) (Figure 3). Given the reliable measurement methods used for satisfaction and the absence of significant downgrading factors, we maintained the certainty of evidence as “high”.

#### 3.4.2. Assessment of Pain After Nursing

A total of five studies have evaluated the level of pain in patients after receiving different types of nursing by visual analogue scale (VAS). VAS was used as the criterion, with a full score of 10 higher scores indicating more severe pain [22,23].

**Figure 4:**
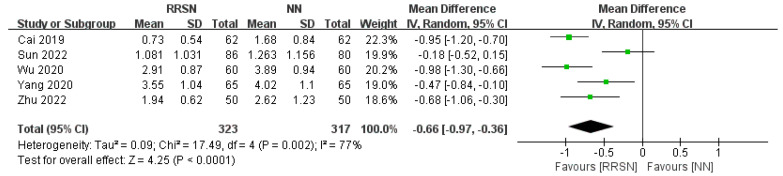
Comparison of visual analogue scale between Rapid Rehabilitation Surgical Nursing and Normal Nursing by forest plot [14,17,18,19,20]. Note: RRSN: Rapid Rehabilitation Surgical Nursing; NN: Normal Nursing; CI: Confidence interval; SD: Standard Deviation. Effect size estimates are depicted by filled squares, with horizontal whiskers corresponding to 95% CIs. The filled diamond indicates the overall mean effect size.

For the assessment of pain following care, due to consistent results across multiple studies, we initially rated the evidence as “high”. However, there was significant heterogeneity (I^2^ = 77%, *p* = 0.002), so we used a random-effects model for analysis. The MD value was −0.66 (95% CI: −0.97, −0.36), *p* < 0.01 (Z = 4.25), indicating that the pain level in the RRSN group after care was significantly lower than that in the NN group (Figure 4). Considering the widely accepted and reliable VAS scale used to measure pain levels, we defined the certainty of the evidence as “moderate”.

#### 3.4.3. Evaluation of Mobility After Nursing

A total of five studies have evaluated the postoperative mobility of patients. Three of the studies were conducted by the Japanese Orthopedics Association (JOA) score in terms of subjective symptoms, sensory impairment, decreased muscle strength, lower limb elevation test, limitation of routine activities, and bladder function. The lower the JOA score, the more severe the decline in spinal nerve function [24,25]. On the other hand, three of the studies used the Oswestry dysfunction index (ODI) to evaluate the lumbar spine function of patients. With a total score of 50, the higher the ODI, the more severe the lumbar dysfunction [26,27].

**Figure 5:**
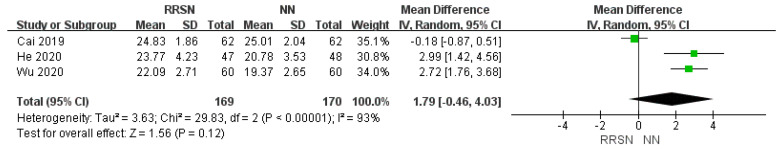
Comparison of Japanese Orthopedics Association between Rapid Rehabilitation Surgical Nursing and Normal Nursing by forest plot [14,16,18]. Note: RRSN: Rapid Rehabilitation Surgical Nursing; NN: Normal Nursing; CI: Confidence interval; SD: Standard Deviation. Effect size estimates are depicted by filled squares, with horizontal whiskers corresponding to 95% CIs. The filled diamond indicates the overall mean effect size.

**Figure 6:**
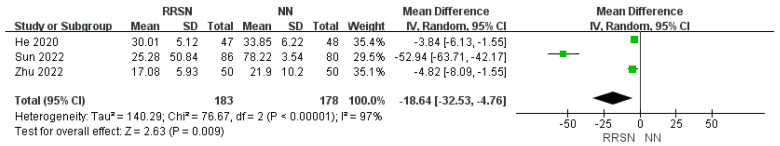
Comparison of Oswestry dysfunction index between Rapid Rehabilitation Surgical Nursing and Normal Nursing by forest plot [16,17,20]. Note: RRSN: Rapid Rehabilitation Surgical Nursing; NN: Normal Nursing; CI: Confidence interval; SD: Standard Deviation. Effect size estimates are depicted by filled squares, with horizontal whiskers corresponding to 95% CIs. The filled diamond indicates the overall mean effect size.

For the assessment of functional capacity after care, due to consistent results across multiple studies, we initially rated the evidence as “high”. However, there was significant heterogeneity in the JOA scores (I^2^ = 93%, *p* < 0.001) and ODI scores (I^2^ = 97%, *p* < 0.01). Using a random-effects model analysis, the JOA score MD value was 1.79 (95% CI: −0.46, 4.03), *p* = 0.12 (Z = 1.56), indicating no significant difference in self-care ability among patients receiving different forms of care (Figure 5). The ODI score MD value was −18.64 (95% CI: −32.53, −4.76), *p* < 0.01 (Z = 2.63), indicating that the disability level of patients in the RRSN group was significantly lower than that of patients in the NN group (Figure 6). Therefore, we defined the certainty of the evidence for JOA scores as “low” and for ODI scores as “moderate”.

#### 3.4.4. Assessment for Psychological Disease Risk

**Figure 7:**
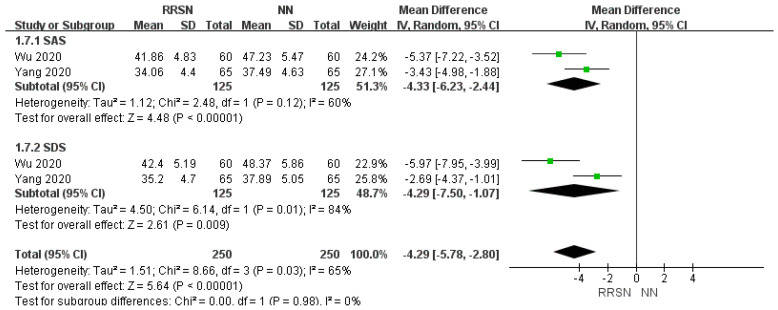
Comparison of psychological disease risk between Rapid Rehabilitation Surgical Nursing and Normal Nursing by forest plot [18,19]. Note: RRSN: Rapid Rehabilitation Surgical Nursing; NN: Normal Nursing; CI: Confidence interval; SD: Standard Deviation; SAS: Self-rating anxiety scale; SDS: Self-rating depression scale. Effect size estimates are depicted by filled squares, with horizontal whiskers corresponding to 95% CIs. The filled diamond indicates the overall mean effect size.

Self-rating anxiety scale (SAS) and Self-rating depression scale (SDS) were used to evaluate anxiety and depression after normal or rehabilitation surgical nursing intervention. SAS or SDS score ≥50 indicated that the patient was accompanied by anxiety or depression, and the degree of the corresponding symptoms was serious with an increase in the SAS or SDS score [28,29] (Figure 7).

**Figure 8:**
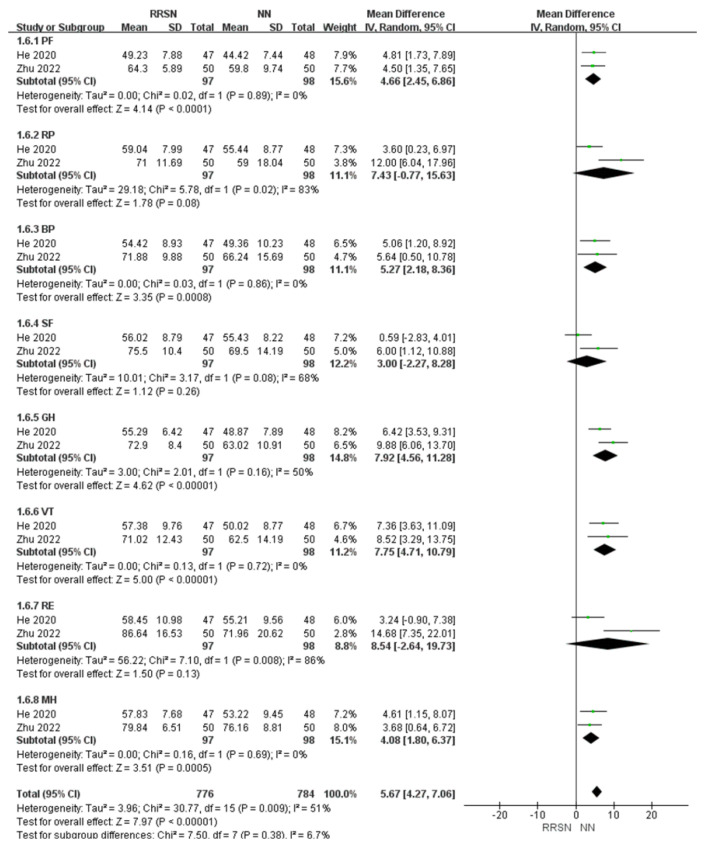
Comparison of 36-Item Short-Form Health Survey scale between Rapid Rehabilitation Surgical Nursing and Normal Nursing by forest plot [16,20]. Note: RRSN: Rapid Rehabilitation Surgical Nursing; NN: Normal Nursing; CI: Confidence interval; SD: Standard Deviation; PF: Physical Function; RP: Role-Physical; BP: Bodily Pain; SF: Social Functioning; GH: General Health; VT: Vitality; RE: Role-Emotional; MH: Mental Health. Effect size estimates are depicted by filled squares, with horizontal whiskers corresponding to 95% CIs. The filled diamond indicates the overall mean effect size.

For the evaluation of the risk of psychological disorders in patients after NN and RRSN interventions using the SAS and SDS scales, due to consistent results across multiple studies, we initially rated the evidence as “high”. However, there was significant heterogeneity in the SAS (I^2^ = 60%, *p* = 0.12) and SDS scores (I^2^ = 84%, *p* = 0.01). Using a random-effects model analysis, the SAS-MD value was −4.33 (95% CI: −6.23, −2.44), *p* < 0.01 (Z = 4.48); the SDS-MD value was −4.29 (95% CI: −7.50, −1.07), *p* < 0.01 (Z = 2.61); the total MD was −4.29 (95% CI: −5.78, −2.80), *p* < 0.01 (Z = 5.64). This indicates that the risk of psychological disorders in patients in the RRSN group was significantly lower than that in the NN group. Therefore, we defined the certainty of the evidence for SAS and SDS scores as “moderate” (Figure 8).

#### 3.4.5. Self-Evaluation of Patient’s Health After Nursing

A total of two studies used the 36-item Short-Form Health Survey scale (SF-36 score) to qualitatively assess patients’ health status after discharge, which includes physical function, role-physical, bodily pain, social functioning, general health, vitality, role-emotional, mental health [30,31].

For the evaluation of patient health status upon discharge using the SF-36, due to consistent results across multiple studies, we initially rated the evidence as “high”. There was significant heterogeneity in the overall studies (I^2^ = 51%, *p* < 0.01). Using a random-effects model analysis, in the SF-36 self-assessment form, there were three data items with no significant differences: RP with an MD of 7.43 (95% CI: −0.77, 15.63), SF with an MD of 3.00 (95% CI: −2.27, 8.29), and RE with an MD of 8.54 (95% CI: −2.64, 19.73). However, after overall analysis, the total MD was 5.67 (95% CI: 4.27, 7.06), *p* < 0.01 (Z = 7.97), indicating that patients in the RRSN group had significantly higher self-health evaluations after care than those in the NN group. Therefore, we defined the certainty of the evidence for SF-36 scores as “moderate”.

### 3.5. Publication Bias

**Figure 9:**
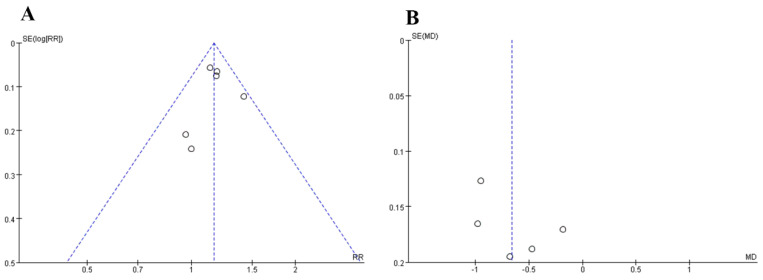
Funnel plots of bias. (**A**): Rehabilitation evaluation; (**B**): visual analogue scale. The horizontal axis represents the effect quantity, and the vertical axis represents the accuracy. Each point represents a study included in the meta-analysis. The blue dashed line represents the distribution of the study effect size when there is theoretically no publication bias.

Draw a funnel chart by comparing the rehabilitation evaluation and the VAS score whether there is publication bias in this meta-analysis. The amount of data on both sides of the dashed line in Figure 9A,B is the same, but not completely symmetrical, and there may be publication bias. Therefore, we performed Egger’s regression test to analyze publication bias. The results showed that the *p*-value for the slope was less than 0.001, while the skewness was greater than 0.05, indicating that the slope is significantly different from zero, but the skewness is not significantly different from zero (Table 2). We cannot reject the null hypothesis, which suggests that there is no publication bias. The reason for this situation may be related to the fact that the sample size selected by some controlled experiments included in the literature is too small, which makes the effect value overestimated and causes bias.

## 4. Discussion

Lumbar disc herniation (LDH) is one of the most common diagnostic causes of lower back pain and a common cause of radiculopathy [32]. Every year, there are 5 to 20 cases per 1000 adults. Minimally invasive lumbar interbody fusion through the intervertebral foramen is one of the treatment methods, which can be completed using small incisions and tubes. Compared with open discectomy, minimally invasive surgery has advantages such as shortened surgical time, reduced blood loss, reduced risk of small joint injury, and reduced postoperative complications [33]. In recent years, the ERAS concept has gradually been applied in fields such as orthopedics and gastrointestinal surgery [34]. By applying a series of intervention measures to improve and shorten postoperative recovery, the goal of accelerating recovery is achieved.

This study included seven RRSNs in the RCT study on postoperative care of lumbar intervertebral disc surgery. By comparing the effects of RRSNs and NN on postoperative pain improvement, self-care ability, psychological health evaluation, health self-assessment, and satisfaction, the study compared the effects of RRSNs and NN on patients.

The evaluation of postoperative patient pain is crucial in the treatment of LDH. A total of five articles used VAS as a pain indicator. VAS is mainly scored based on the professional evaluation of medical staff, which is more objective and professional, and is also an easily quantifiable indicator [35,36]. All five articles indicate that RRSN can significantly reduce patient VAS, which is consistent with the analysis results of this study. The patient’s satisfaction with nursing care and self-assessment of health also reflect to some extent the patient’s subjective judgment of postoperative pain. Similarly, based on the patient’s subjective judgment, RRSN has a better rehabilitation effect on LDH. LDH patients often experience obvious pain symptoms such as lower back and leg pain, sciatica, etc., which greatly affects their quality of life. Through pain care, various measures can be taken to alleviate the patient’s pain and help alleviate discomfort and pain [37].

Effective pain management can not only help patients alleviate pain but also assist them in better rehabilitation training and activities. Reasonable pain management can reduce patients’ pain perception during the rehabilitation process, and improve their motivation and participation in sports. Among the seven articles, three evaluated the patient’s self-care ability by analyzing the JOA score. Meanwhile, three of them evaluated the patient’s disability level by analyzing ODI. Through statistical analysis, it was found that there was no significant difference between RRSN and NN in the evaluation of self-care ability. This may be due to the improvement in activity ability being influenced by various factors, including individual differences, the duration and intensity of rehabilitation training, and the subjective nature of the JOA score itself. Different researchers and assessment methods may also contribute to inconsistencies in results. At the same time, the judgment of self-care ability may also be influenced by the patient’s psychological state [38]. The evaluation of the degree of disability in patients is more objective. Other literature has also evaluated the patient’s mobility, but the data have not been well quantified and are not suitable for meta-analysis. However, the research results also demonstrate the superiority of RRSN.

The reduction of pain can also alleviate the psychological burden on patients, provide emotional support, maintain diagnosis and treatment enthusiasm, and improve their quality of life [39]. Wu and Yang’s study evaluated the risk of psychological disorders in LDH patients using SAS and SDS indicators, and the results showed that RRSN can significantly reduce the risk of depression and anxiety in patients. Zhu’s research also analyzed the psychological status of patients, mainly scoring their emotions and psychological status through survey questionnaires. Although the data from these two studies do not match, the results also demonstrate the improvement effect of RRSN on the psychological status of patients.

Similarly, most of the postoperative rehabilitation items are also included in the patient self-test form. Although the health self-assessment form is not objectively evaluated by professional medical staff, it reflects the patient’s subjective recognition of the improvement of their disease after receiving different nursing care [40]. RRSN has advantages in terms of mental health, pain relief, and physical mobility.

In this study, some outcomes (such as pain evaluation and psychological disease risk assessment) showed high heterogeneity (I^2^ > 50%). This high heterogeneity may be due to several factors: (1) Differences in study designs, including sample size, study locations, and intervention durations, which can lead to heterogeneity in results. (2) Variations in participant characteristics such as age, gender, and disease duration, which can also contribute to heterogeneity. (3) Differences in the specific implementation details of RRSN, with varying interventions across studies, which can result in heterogeneous outcomes. (4) Differences in assessment tools and scoring criteria used in various studies, can also lead to heterogeneity. For example, pain assessment used VAS scores, while psychological disease risk assessment used SAS and SDS scores.

These results suggest that in practical clinical applications, multiple factors need to be considered, including the specific circumstances of patients, personalized rehabilitation training needs, and the actual effectiveness of rehabilitation care. Although RRSN performs better in most cases, there may be a need for further optimization and adjustment of care strategies in certain specific situations. Despite some publication bias and heterogeneity, the main outcomes still support the effectiveness of rapid recovery surgical nursing (RRSN) in improving postoperative recovery in patients with Lumbar disc herniation (LDH). Due to the inclusion of only seven pieces of English literature in this study and the lack of relevant quantitative data in the included literature, a meta-analysis cannot be conducted. As for the improvement effect of RRSN on LDH, more clinical studies need to be accumulated in the future for analysis.

## 5. Conclusions

In summary, RRSN intervention for patients with Lumbar disc herniation has important value, which can alleviate pain symptoms, improve their psychological state, help restore lumbar function, and also improve patient satisfaction and quality of life. It is worth promoting.

## Figures and Tables

**Table 1:** Basic characteristics of the included studies.

	Year of Publication	Sample Size	Age	Nursing Methods	Outcomes Indicators
		C (Male, Female)/T (Male, Female)	C (Mean ± SD)/T (Mean ± SD)		
Cai [14]	2019	62 (39, 23)/62 (35, 27)	61.15 ± 9.51/60.78 ± 9.85	Stepped nursing intervention/routine nursing intervention	Satisfaction evaluation, pain evaluation, and action ability evaluation
El-seadi [15]	2022	30 (18, 12)/30 (19, 11)	39.57 ± 8.12/40.97 ± 7.92	Protocol of care designed by the researcher/Routine hospital care	Satisfaction evaluation, and pain evaluation
He [16]	2020	48 (27, 21)/47 (25, 22)	45.88 ± 4.99/46.01 ± 5.12	Continuous nursing based on wechat platform/routine continuous nursing	Satisfaction evaluation, action ability evaluation, and patient health self-assessment
Sun [17]	2022	80 (35, 45)/86 (27, 59)	58.86 ± 10.88/56.92 ± 11.70	Enhanced recovery after surgery (ERAS) care/traditional perioperative care	Satisfaction evaluation, pain evaluation, and action ability evaluation
Wu [18]	2020	60 (32, 28)/60 (33, 27)	59.18 ± 12.65/59.47 ± 12.53	Stepwise rehabilitation nursing/routine nursing	Satisfaction evaluation, pain evaluation, action ability evaluation, psychological disease risk assessment, and Sleep quality evaluation
Yang [19]	2020	65 (31, 34)/65 (36, 29)	47.90 ± 6.20/48.70 ± 5.40	Routine nursing and rehabilitation nursing (RN) mode/routine nursing mode	Pain evaluation, psychological disease risk assessment, and evaluation of postoperative complications
Zhu [20]	2022	50 (30, 20)/50 (24, 26)	55.30 ± 14.09/53.58 ± 12.79	Rehabilitation training on the basis of routine functional exercise/routine functional exercise	Satisfaction evaluation, pain evaluation, and action ability evaluation patient health self-assessment

**Table 2:** Egger’s regression test to test hypothesis.

Std_Eff	Coef	Std. Err.	t	*p* > |t|	[95%Conf. Interval]	
Slope	2.341536	0.5815782	9.34	0.000	3.234875	6.254123
Bias	−0.3556142	0.4123597	−0.58	0.627	−1.387412	0.9641239

## Data Availability

The raw data supporting the conclusions of this article will be made available by the authors upon request.
